# An Inquiry into the Opinions, Ancient and Modern, Concerning Life and Organization

**Published:** 1822-09-01

**Authors:** 


### Medico-Chirurgical Review,

AND

### JOURNAL OF MEDICAL SCIENCE.

(Analytical Series.)

"Nec Aranearum textus ideò melior, quia ex se fila fingunt; nec noster vilior, quia ex alienis libamus, ut apes."

Vol. III.]  SEPTEMBER 1, 1822.  [No. 10.

### I.

*An Inquiry into the Opinions, ancient and modern, concerning Life and Organization.* By John Barclay, M.D. Lecturer on Anatomy and Surgery; Fellow of the Royal College of Physicians, &c. &c. of Edinburgh. Octavo, pp. 542. Edinburgh, 1822.

Our readers will not accuse us of much propensity towards metaphysical disquisitions. Our reasons for abstaining, in general, from such lucubrations, are founded on a conviction that, in all investigations respecting the *nature* of life, and the *destiny* of the soul, the fool and the philosopher—the cobbler and the craniologist—the merry-andrew and the metaphysician, start upon perfectly equal terms—with this exception—that the creed of the philosopher is always more absurd than that of the fool. The truth of this position, paradoxical as it may appear, will, we apprehend, be made abundantly manifest in the course of this article. Discussions on life and organization have, of late, occupied so much the attention of the public, that we are reluctantly drawn into the vortex—yes, a *vortex* or eddy, *off* the general stream of medical science, in which, however long we whirl about, we can never make one foot of progress. We shall invariably come back to the point from whence we started—few of us, we fear, made much better or wiser during our circumgyrations! We sincerely wish indeed, that physicians and physiologists, at least, had confined their researches to the laws which govern and the diseases which assail the human body, leaving the mortality or immortality of the soul to philosophers, metaphysicians, and divines:—or, if they *did* enter into the investigations of mind, that they would acknowledge their own ignorance when their faculties could go no farther, instead of dogmatically pronouncing in the negative, on doctrines generally received by mankind, and which appear necessary to the welfare of society, as well as the peace and happiness of individuals. No human being on this earth ever had, or ever will have, an atom of proof, (independent of Revelation,) respecting the nature and ultimate destiny of the soul. Those, therefore, who deny its future existence, are just as dogmatical and presumptuous as those who maintain it—with this difference, that they broach a doctrine repugnant to the best feelings of mankind, and utterly subversive of all those checks and restraints, which reason, religion, and morality, would impose on the turbulent passions of our nature. If we are placed here irresponsible to any other tribunal than those of human creation, we must be dolts indeed to curb even our most vicious propensities, farther than what may just keep us clear of the gallows! Nay, if the indulgence of a crime be thought to convey more pleasure, than a swing at Tyburn pain, we know not on what principle the Materialist should hesitate to suffer the one, for the enjoyment of the other. In fact, this is the doctrine precisely, on which the most abandoned of the human race act every day ;—it is the doctrine which is too often instilled into the tender minds of medical youth along with their physiological knowledge—it has been publicly taught in our schools—boldly pronounced in our orations—and fearlessly published in our books! How is this to be remedied? Certainly not by attempting to prove the truth of contrary doctrines; for, as we said before, we have no proofs, pro or con, but in REVELATION, and this will not be listened to by the Materialist. The only remedy, in our humble opinion, is that which Dr. Barclay has adopted—namely, an exposition of the absurdities, contradictions, and chimæras, of the philosophers themselves. It will be seen in this exposition, too, that the reigning doctrines of the day, among our modern philosophers, are nothing but new versions—indeed, generally but mere repetitions of the visionary speculations engendered in the brains of their predecessors, even from the earliest ages. Stripped of their novelty, and devoid of solid foundation, as all such speculations must ever be, these doctrines will, we hope, gradually sink once more into oblivion, and men, feeling their own ignorance, and seeing the presumption of these illuminati, will come back again to the sober path of reason, and the steady light of religion and virtue. It is lamentable, however, to think that, some men, whose talents, genius, and acquirements, might have enabled them to diffuse the blessings of science and the charms of literature over a grateful land, have chosen, with fiend-like satisfaction, to shine only to mislead—to flash only to destroy. "Their beams," says an able writer, "are a beacon set up by the Genius of Evil—a beacon that would warn us *from* that which is *safe*, only to decoy us *to* that which is *dangerous*—having a false light to amuse, a syren to allure, a Circé to intoxicate; lest we should perceive, that the fatal coast is covered with wrecks."* Under these circumstances, we hope we shall be excused, if not commended, for dedicating a very considerable portion of our present number to the review of a work, which is calculated to improve the humble by the profundity of its erudition; and repress the presumptuous by the poignancy of its satire—to strengthen the good man's belief in a future state of existence, and weaken the sceptic's overweening confidence in the powers of his own unassisted reason.

The analysis of a work like this, is not an easy task; and we are sure the worthy and learned author will excuse us, if we frequently endeavour to convey the spirit of a passage in our own language, rather than in an extract, since habit has, we think, given us the power (a very humble pretension) of sometimes lessening the number of words, without weakening or obscuring the sense.

In a short preface to the work before us, Dr. Barclay informs us of the reasons which gave rise to his present inquiry. These were, the differences of opinion which exist respecting the nature of man—some thinking that such a mechanism may have been produced without a divine constructor—others not. The object of the present work, therefore, is to state the arguments on both sides—to examine their legitimacy and force—to offer his own opinion—and then leave the reader to judge for himself.

As young men, our author justly observes, are naturally inquisitive respecting the laws of organization, and are frequently led to form hypotheses, on slender grounds of information, an inquiry like this may be useful to them, by shewing them what ingenious and learned men have written on the subject. It may probably tend to moderate the excesses of vanity, and prevent them from betraying their ignorance, by publishing as new, "opinions which have repeatedly been published before—have repeatedly been obsolete—been repeatedly revived—and repeatedly become obsolete again." On this subject, he observes, few original opinions or arguments upon either side, have been advanced since the days of Lucretius, or even of Aristotle. The novelties are chiefly those of expression or manner, with some attempts at illustration by aid of the microscope and chemistry; but the im-

* Coulton's Remarks on the Talents of Lord Byron.

pelling motives, the leading opinions, the chief arguments, have scarcely suffered any change through a series of centuries.

The work is divided into four chapters, each subdivided into numerous sections. The first chapter contains the philosophical and popular opinions of the ancients concerning the nature and variety of animating causes, and the principal arguments employed to prove that these causes originate in matter:—the second chapter gives an account of some very vague and general terms employed in physiological investigations:—the third is on the opinions of those modern physiologists who ascribe the phenomena of life to mechanism and the effects of chemical affinities:—the fourth exhibits the opinions of some distinguished ancients and moderns who have ascribed organization and the other vital phenomena to an internal animating principle.

Chap. I. *Sect.* I. In all languages there are terms to express those two remarkable states of organized bodies—the *living* and the *dead*. The clown can distinguish these as clearly as the chemist. There is a kind of intermediate state, in which plants and animals are occasionally, and their seeds and eggs generally, found. In these cases the obvious functions of life are partially or totally suspended—but the suspension does not, as in the dead state, arise from derangement of the system—it wants only some auxiliary agents, as heat for instance, to bring the powers into play. The living state is the most remarkable of all, and has naturally excited the attention of philosophers in every age and country.

*Sect.* II. This exhibits a very summary view of ancient opinions respecting vital phenomena. The Greeks, in general, ascribed these phenomena to the *psyche*—in Latin, *anima*—in English, *soul*, or vital principle. Their opinions, however, were very various. Democritus, Epicurus, and the Stoics, thought it corporeal, but could not agree as to its substance. The Stoics asserted that it was warm ignited air—Hippo, that it was water—Democritus, that it was fire—Heraclitus, that it was a vapour or exhalation from the body!

The notions of those who believed the soul to be incorporeal, were, if possible, more absurd than those of the Materialists. Thales maintained that it was always in motion, and itself the cause of motion—Pythagoras, that it "was a self-moving monad"—Plato, that it was something conceivable only by the understanding, moving according to harmony—Aristotle, that it was a fifth essence or element distinct from any of the other four—others, that it was an emanation from one universal soul, or anima mundi. It is needless to load our pages with the doctrines of transmigration and the plurality of souls—as the opinion of Plato, for instance, that there was a soul for the belly, another for the chest, and a third for the head. We shall therefore be very concise with the *third section*, on different souls in the same body. This opinion became very early prevalent. Empedocles gave a rational and sentient soul to every animal—the first derived from the gods, the second from the elements. The vulgar creed that ghosts or apparitions are sometimes seen near the place where the body lies, is well known. Lucretius, though he admits the existence of these *simulacra*, asserts that they are mere pellicles or membranes cast off from the surfaces of bodies, like old sloughs from grasshoppers.

"Quæ, quasi membranæ summo de corpore rerum

"Dereptæ, volitant ultro, citroque per auras."

This is a pretty early specimen of the credulity of Sceptics.

*Sec. IV. Pre-existence of Souls.* The notion of a *future* existence, however it may have got originally into the mind, seems, from the very beginning of the human race, to have constantly followed them wherever they emigrated or settled—nay, it even seems to have sprung, as it were, spontaneously, in every soul the least elevated above that of a brute. The idea of a *pre-existence*, however, has been limited chiefly to contemplative minds engaged in philosophical research. It occurred to Plato, who supposed the soul to be different from the body, and its innate ideas but the reminiscences of former impressions. It occurred to Pythagoras and the Eastern sages, who believed in transmigration; and it occurred to all who believed that substantial entities are eternal, and that thought and sensation are not to be considered as the accidents of matter.

*Sect. V. Future Existence.* The opinions among the ancients, respecting this state, were almost without number. Some doomed the soul to wander about without a body; but as that state was difficult to conceive, it was generally allowed some kind of tabernacle, or at least some kind of form. Thus equipped it was sent to the clouds—to the stars—to happier regions above—to regions in the bowels of the earth—or through other animals, in a series of transmigrations. Such were the various opinions of the ancients concerning the soul or vital principle. We do not conceive that these opinions make either for or against the existence, present or future, of a soul. Those who would deny its existence because those who believe in it are not agreed in opinion, might, upon this principle, reject almost every branch of philosophy.

*Sec.* VII. *Organism.* All opinions respecting the vital phenomena have rested on one of two bases—a certain organism of the materials of which the visible structure is composed—or a principle totally distinct from matter, receiving a distinct appellation in all languages.

The observations and reasonings which lead to organism or materialism, say its supporters, are not only natural, but the conclusion almost unavoidable. To what do we owe the difference of sounds proceeding from the flute, the violin, the harp, but to difference of structure—to what can we attribute the difference of functions in the animal system, as seeing, hearing, smelling, but to differences of structure in the organs by which they are displayed?

An egg, say they again, does not exhibit any thing analogous to vital phenomena. The eye cannot trace in it any thing but an organized structure. In this state, regulate the temperature so as to prevent the derangement of its parts, and it will continue for months or years in the same inert condition. But after these years apply the degree of heat which the mother communicates while hatching, and the embryo within will begin to grow, to move, to live—and at last will exhibit all the instincts, appetites, and passions belonging to the species that first gave it birth. Observe, say they, the swallow or other hybernating animal. It grows torpid and apparently dead in the winter. On the return of warmth we see it begin to move, to live, to seek a mate, and propagate its kind. What is there here, they ask, but organism and heat? If there be any other principle, who is the man that has seen it, heard it, touched it, or tasted it? No man. All other causes, then, are merely imaginary. After seeing what we have seen, let us remain content with the obvious causes, and leave enthusiasts to hunt after mysteries, or indulge in fanciful hypotheses about spirit, and life, and soul.

Should it be asked what is the cause of those processes of organization which produce the organism that produces life—the answer is natural, easy, and obvious. "It is," say they, "a number of the particles of matter of certain kinds, in certain proportions, and in certain temperatures, combining together by their chemical affinities." Mighty clear certainly! After stating a few of the many wonderful combinations and changes effected by chemistry, they ask us to ascend yet a little higher, and mark the *animalculæ infusoriæ* that spring up in myriads in vegetable infusions—the *fasciolæ hepaticæ* growing in the livers of sheep—the various *vermes* in the intestines of man—the thousand species of *lice*, each peculiar in its own species of animal or plant—all without origins, without parents, without sexual intercourse. View these facts candidly, say they, and tell us to what you can ascribe living organized systems of matter, but to chemical processes.

"In what other light can we possibly view the sexual intercourse than merely as a circumstance which is often necessary to favour these processes at their commencement? Nay, in what other light can we possibly see even man himself than as a species of chemical compound, whose particles at the last hour of dissolution must again return each to the element from which it came; while the fabric they composed, like the flower of a season, or the insect of a day, after leaving perhaps another in its stead, shall, with all its instincts, appetites, and passions, and with all its reasoning and boasted powers, that were the result of its temporary organism, perish for ever?" P. 30.

That such were the commencement and such the termination of human existence, many may wish rather than believe—and a few may believe rather than wish; but those accustomed to reason and reflect, will not place implicit reliance on observations that are carelessly made, and assertions that are so confidently uttered. Even granting that there is nothing in the universe but matter, where are the *kinds* that organize animals and plants—where the *qualities* by which they organize particular structures? We can readily comprehend how the same materials, whether earth, wood, or stone, may be made to assume an infinity of forms; but the materials have nothing to do with the organization. It is at this day invariably acknowledged that every part of an animal or plant dug up from the bowels of the earth, must once have lived—and that some one of the vital phenomena must have preceded and even been necessary to its organization. It seems, therefore, to be the natural and the necessary conclusion, that so far is organism from being the *cause*, that it is rather the *effect* of vital phenomena; and hence it follows that the materials of food and drink, like the kinds of materials composing an automaton, may enter into myriads of structures, having no more concern in regulating their size, proportions, or forms, than the timbers have in devising the plan of a ship, into which they may enter—with all their chemical affinities it is true—but still merely as the materials, and not as the architects or contrivers.

In respect to the seeds of plants and eggs of animals, as they are evidently as much organized structures, and as much the effects of vital phenomena, as the plants and animals from whence they sprung, it is but a silly and puerile sophism to first represent them as extremely minute chemical particles, and then, taking advantage of their chemical affinities, say that they afterwards produce animals and plants. The fact is, they are animals and plants themselves in their incipient state of existence—and this sophism, if closely examined, means no more than that plants and animals, however young and diminutive at their commencement, grow older by time, and larger by nutrition.

We must pass over the succeeding three or four sections on the "origin of things," as explained by Anaxagoras, Ocellus Lucanus, Democritus, and others, in order that we may come at once to the great arch-Atheist and Materialist of antiquity, LUCRETIUS, the expounder of the atomic doctrines of Epicurus. This poet embraces with enthusiasm the views, and opposes the enemies of Epicurus. He combats every objection with all the acumen of the logician, and the animation of the poet. He pours forth his whole genius and talents to illustrate, support, and recommend the favourite atoms of his favourite philosopher.

Yet with all the confidence which he has in these atoms, he never brings them to the test of observation or experiment. He never pretends that he has seen them, or indeed that they can be seen. He bestows on them many and various shapes —but never explains how their shapes co-operate to form an animal or plant—nor how, amidst all their various combinations, they never by chance light upon any thing in structure, materials, or external form, exactly resembling any work of art. He asserts that, even in his time, many animals were formed by showers and sunshine out of the mud.

"Multaque nunc etiam existunt animalia terris,
"Imbribus et calido solis concreta vapore."

Here the showers and sun must have been the father, and the earth the mother, of these new animals.* But how come these parents to bring forth young so unlike their kind?—why did they not bring forth young earths and suns? Instead of answering these questions, the poet is only busied in accounting why mother Earth was not so fruitful in his time as she had been formerly. And what were the reasons?—very clear and natural ones. "The times of child bearing,"

* —— "E terra quonian sunt cuncta creata."

says he, "are always limited; and being old and exhausted, these periods in mother Earth were approaching to a close."

> "Sed, quia finem aliquam pariundi debet habere
> "Destitit; ut mulier, spatio defessa vetusto."—*Lib.* v.

Yet, to another question, how came the world so advanced in age, to be such a novice in the arts and sciences? He replies, somewhat forgetful of his former statement, that, from the little progress which it had made in the way of invention or improvement, it must certainly have been of very *recent origin* at the time he wrote—in fact, only in the course of its education!

> "Verum, ut opinor, habet novitatem summa recensque
> "Natura mundi est; neque pridem exordia cepit.
> "Qua re etiam quædam nunc artes expoliuntur;
> "Nunc etiam augescunt." *Lib.* v.

It appears from Lucretius that Mother Earth was but a bungling artist herself at first, producing monsters of very singular appearances, incongruous forms, and with unnatural adhesions, &c. A superior authority therefore interposed, and took the management out of her hands. This Being, who belonged neither to atoms nor to elements, has been a very convenient substitute for a God, both in ancient and modern times. Her name is NATURE—a singular personage of the feminine gender, that is always generating, like Mother Earth, but is never seen generating after her kind. She is invested by our modern Atheists with extensive influence, incessant activity, and uncommon prudence, having obtained the direction of the atoms and elements in all their operations, seeing into futurity, preventing what she does not approve, creating and bringing to perfection all living things—and all this agreeable to *laws* imposed upon her by a higher power, which some call *Fate*, and others Necessity. In this part of the theory, Lucretius seems to have lost all confidence in the eternal rambling of his atoms. He thinks now that all must be regulated by diversities of *seeds* or *organic particles*, endowed each with a peculiar *secreta facultas* that makes them both living and organic. He deduces the soul also from a seed, and makes it display in its growth and evolution the species of seed from which it had sprung. Hence he concludes that it is from radical difference in the faculties of the soul, and not difference of organization in the body, that the lion is fierce, the fox crafty, and the stag timid.

As for the opinion that the soul or animating principle organized the body, Lucretius rejects it—*because* he cannot comprehend how it could be.

———At, qua possint, via nulla *videtur*:

Haud *igitur* faciunt animæ sibi corpore et artus.

Aware, however, that he might fairly be asked how his *semina* were originally organized, he is sadly perplexed, and merely informs us that "heat and air and the invisible power of the wind, being mixed with that *active principle* that distributes motion and sensation to them all, they together constitute but one nature." As for the origin of the active principle, he confesses that it is utterly concealed in the innermost recesses of the body, whence it is latently diffused through every member, being the soul of the soul, the strength of the mind, and the energy of life—in short, that it reigns as a sovereign throughout the body, and *is without a name*.

"Sic tibi nominis hæc expers vis, facta minutis
"Corporibus latet."

It is to this *nameless something* we owe the pulsations of the heart, the sensitive powers of the organs, the pleasure or pain felt in the nerves or bones.

Lucretius observed of old, (as the philosophers have observed in our own times,) that as the world had been long in a state of terror or dread of incorporeal beings, the principal object in writing was to rescue mankind from their vain apprehensions, by shewing that all things are corporeal and composed of atoms—that those atoms will in time separate and be dispersed—and that even the soul shall cease to exist or feel. He seems anxious to inculcate the notion that the world has been arranged, and the organism of plants and animals constructed, without any view to their functions or uses, "and so men," says he, "should avoid the error of supposing that the eyes were made to see, the feet to walk, &c." No, says he, the organs existed *before* they were used—consequently they suggested the uses, and no uses could have been previously designed for the organs. He distinctly saw that a contrary opinion would lead to an idea that the works of nature, like the works of art, must be the effects of design and intelligence—an idea that would be utterly subversive of his atheistical hypothesis. The absurdity, however, of the doctrine that the organs suggested their own uses, is too obvious to require a word. The credulity, too, of this arch-Sceptic is only equalled by that of some of our modern Materialists. Thus he cannot believe in the immortality of the soul; but he believes in the existence of *simulacra*, thrown off from the surfaces of bodies, and continuing in separate states, constituting ghosts. Again, he strenuously endeavours to prove that, from their inconceivable minuteness, the first principles of things are *invisible;* yet he confidently ventures to determine their shapes, properties, magnitudes, &c. In a moment afterwards, he is such a stickler for the evidence of the senses, that all are enthusiasts or dotards who trust to any thing else! "In this miserable shuffling," says Dr. Barclay, "he is often followed by the modern Materialists, who, without producing the evidence of sense for their own hypothesis, arrogantly demand such evidence for whatever hypothesis is opposed to theirs." We do not agree, however, with Dr. Barclay, in supposing that Lucretius, because he reposed no hopes on the promises of Religion, "could only view it through the medium of fear"—and that therefore "his whole reasoning is employed to remove its impressions." We believe that the great body of the Sceptics, both of ancient and modern times, had, and continue to have, neither hopes nor fears. Indeed, we do not see how a cultivated mind, that has no hope in the promises of revelation, can have any dread about futurity. They have a curse attending them, however, which is perhaps far worse than the occasional terrors of the religious enthusiasts. It is that melancholy, dreary, depressing prospect of *annihilation*, which intrudes itself constantly on their thoughts, damps every enjoyment of *this* life, and precludes, of course, all anticipation of another! There is not a Sceptic on this earth who does not envy the *terrors* of the most ignorant fanatic—*because* he well knows that with those terrors are mingled bright hopes and cheering illusions (if indeed illusions) of another world. These are excitements which can never thrill the cold heart of the Sceptic. His soul is like the stagnant lake—it is never agitated by

——— "That pleasing hope, that fond desire,
"That longing after immortality!"———

which prevents life from becoming insipid. He is, in fact, an object to be pitied, in more senses than one—to be pitied if he be in the *wrong;* on account of that futurity which he disbelieves—to be pitied if in the *right;* because, in this case, if in any, "ignorance is bliss," and it is manifestly "a folly to be wise."

We can form some excuse, however, for the apparently honest indignation which Lucretius expresses against the religion (superstition) and priests of his time, when we consider how much the people were immersed in ignorance, and the priesthood in knavery. We must also confess, with shame and sorrow, that religion, or rather superstition, has too often, even up to the present moment, been perverted to the worst of purposes, by the designing, fanatic, ignorant, and tyrannical part of the world. The notions, as Dr. Barclay justly observes, of a Deity, of a watchful Providence, of a future state of rewards and punishments, may be, and have been, *perverted* to the worst of purposes. Instead of producing peace upon earth, they have too often been made the pretence for drawing the sword, for setting the son against the father, the daughter against her mother, and for making a man's household his foes.* But surely, as our author properly adds, the perversion or abuse of a principle can never, in the eye of genuine philosophy, be made an objection to the principle itself. The alternative which is here recommended, of disregarding all notions of religion, was observed by the greater part of the ancients to be attended with still worse consequences than any of the systems of Pagan theology. Epicurus and Lucretius themselves acknowledge that some notion of divine beings is quite irresistible, and that it will spring up in the human mind, as a thing indigenous, without the adventitious aid of education. "Intelligi necesse est, esse Deos, quoniam insitas eorum vel potius innatas cogitationes habemus."—*Cicero.* The attempt, therefore, to eradicate this notion from the human race is foolish, mischievous, and must, in the end, prove abortive.

We must pass over entirely the *second* chapter of our learned author's work on nature, the elements, forms and qualities, chance, fate, necessity, matter, &c. &c. and also a great portion of the third chapter, detailing the opinions of Paracelsus, Fray, Darwin, Leibnitz, Priestly, Haller, Buffon, Needham, Maupertuis, Robinet, Blumenbach, Gassendi, Cuvier, Lawrence, and Cabanis, respecting life and organization. We cannot afford space for the notice of more than a very few of these writers.

Blumenbach.—The idea of an eternal being, omnipotent, and omniscient, who watches over the affairs of men, continues their existence beyond the grave, and renders them accountable for their actions in this world, is not equally cherished by all. It is, however, a very general idea, and seems so instinctively implanted in our nature, that no devices have hitherto completely eradicated it from the minds of men—perhaps even of the most sceptical. That there is some invisible agent who superintends the universe all are willing to allow—but all are not willing to attribute to this agent in-

* Matthew, Chap. x.—34.

telligence and moral attributes—or that he will make them responsible for their motives and actions. They are therefore inclined to fancy other agents more suitable to their ideas, or perhaps to their wishes, such as Nature, Fate, Chance, Necessity, or certain invisible energies or powers diffused throughout space, which operate methodically, yet without intelligence or moral attributes—and, if not *material*, are traceable to no other cause that either is or ought to be named. It is to one of these abstract powers that Blumenbach has ascribed the organization of animals, bestowing upon this child of his fancy some peculiarities which he thinks ought to recommend it by their novelty. This power he denominates *nisus formativus*, which presents little novelty to those who have heard of the *vis formatrix* or *nisus* of Kempt, or *formative propensity* of Darwin. The new information conveyed in this hypothesis of Blumenbach amounts to no more than this truism, that—*a plant or animal is formed by the power that formed it*! It is, in fact, recurring to the old and obsolete mode of explaining phenomena, by which creation was ascribed solely to a *vis creatrix*—generation to a *vis generatrix*—concoction to a *vis concoctrix*, &c. What does Mr. Hunter explain by his stimulus of death—stimulus of necessity—stimulus of cessation, &c. His most enthusiastic admirers can hardly pretend to understand him.

But Blumenbach pretends to have ascertained the laws by which his formative nisus operates. What are these laws? The jet of them is—that young animals grow faster than old ones—that the processes of organization are more rapid in the class of mammalia than in oviparous animals—and that in both classes there are certain organs, for instance, the brain, which are generally found to be more regularly organized than others. In his own language the first law is that—"the activity of the nisus is in an inverse ratio to the age of the organized body"—the second that—"the formative nisus is much more active in the embryos of mammalia than in those of oviparous animals"—the third that, "in the formation of some particular parts of an organized body, the formative nisus is much more regular in its process than in that of others."

There is this apparent novelty, however, in Blumenbach—his attempt to consider a power as distinct from the agent in which it resides, or from which it proceeds—of giving a name to this power, and yet refusing a name to the agent which exercises it. Thus, he gives us to understand that this nisus is a power deriving its origin from some occult quality; and, that this *quality*, though totally *unknown*, has, nevertheless, been found to be a quality of *matter*. If we ask, of what matter is it a quality? Blumenbach admits that it is not a quality of matter in general—that it is confined to organized bodies—and to be found in those bodies *only*, when in that state which is termed *living*—that it seems to depend on the powers of *life*—that it is a quality of *living* bodies, but as distinct from the other qualities of living bodies, (sensibility, irritability, contractility,) as from the common properties of dead matter! After this circle of contradictory and unmeaning reasoning, we may take leave of Blumenbach. If he had simply and candidly confessed that he knew nothing at all about the matter, he and many other philosophers, would have shewn their wisdom in exposing their ignorance!

Cuvier, rejecting the hypothesis of pre-existing germs, indestructible monads, organic particles, and semina rerum, ascribes the organization of plants and animals, sometimes, to what he terms a *vital force;* sometimes, a *vital impulse*—sometimes, to a *species of motion*—and, sometimes, to *life itself*. To a person not blinded by some hypothesis, some distinction between force, impulse, life, and motion, would certainly have occurred. To him, however, it has not occurred. With respect to *life*, he thinks not of deciding whether it be the cause or the effect of motion—he imagines it to be sometimes the one, and sometimes the other. And yet, as Dr. Barclay shrewdly observes, Cuvier must have seen varieties of motion which never produced any thing like life, while, on the contrary, he never could have observed and fully ascertained the presence of life in any one body, in which that life did not exhibit some phenomena of motion proceeding from itself, and originating in itself, independently of any thing like external impulse. In short, Cuvier's explanation of life and organization is, as Dr. Barclay remarks, "nothing but a heap of words; or, to borrow a term from the language in which it is written—mere *verbiage*." After this gentleman's assertion that the laws which regulate living bodies are not only different from those which regulate mere matter, but act in a manner *entirely contrary* to those laws, we could hardly fail to be surprized at his singular warning, *not to consider those two kinds of laws*, *though entirely contrary*, *the one to the other*, *as being absolutely of a different order!* The truth is, Cuvier, like those physiologists who would have the stomach to be any thing but a stomach, is strangely bewildered amidst the confusion of his ideas in labouring to prove that the cause of life may sooner be any thing than what men in general feel and suppose it to be. At one time he says that be life what it will, it cannot be what the vulgar suppose it, a particular principle (principe particulier.) In another place he acknowledges that life can proceed only from life. (La vie ne naît que de la vie.)—Then again he considers it an internal principle, (un principe interieur d'entretien et de reparation ;) and last of all, says (what Mr. Lawrence has since repeated verbatim) that life consists in the sum total of the functions. "Il consiste dans *l'ensemble des fonctions* qui servent à nourrir le corps, c'est a dire la digestion, l'absorption, la circulation, &c." Thus he makes life a cause which owes its existence to its own operations, and consequently a cause which, had it not operated to produce itself, had never operated nor existed at all! Such are the brilliant conceptions and lucid explanations of our modern materialists!

"In concluding these remarks," says Dr. B. "on Cuvier's ideas of the nature of life and organization, it may well be said, that, were any person capable of supporting, with any tolerable degree of success, the doctrines of materialism, it would certainly be Cuvier. He is a naturalist, a chemist, mineralogist, a most distinguished and illustrious anatomist, and has probably examined a greater number of organized bodies than any other individual in Europe. His industry, at the same time, his learning, his eloquence, and his various talents, are of the first order, except his logical acumen, which seems to have been either originally defective, or to have been afterwards miserably blunted by his hypothesis. It is truly pitiful to think of a man with so many endowments, natural and acquired, driven as if blindfold by the fashion of the times, a contemptible vanity, or some wretched inclination, endeavouring to support with all his energy the extravagant idea that the phenomena of design and intelligence displayed in the form and structure of his species, might have been the effects of some antique force, impulse or motion, or of some group of functions, as digestion, circulation, respiration, transpiration, and excretion, which have accidentally happened to meet without any assignable cause to bring them together, to hold them together, or to direct them." 330.

LAWRENCE.—In our notice of this section we shall suspend all sentiments of our own, and adduce nothing but what is found, both in letter and spirit, in the work before us. We shall not even use our own language upon this occasion, but adhere most strictly to the very words and expressions of Dr. Barclay. Mr. Lawrence, whom we esteem, and Dr. Barclay, whom we respect, shall have no cause to say that we have aught extenuated, or "set down aught in malice," on the present occasion.

"Mr. Lawrence," says Dr. B. "a skilful surgeon, a learned physiologist, and a distinguished anatomist, in treating of life and organization, has seldom ventured beyond the steps in which Bichat, Blumenbach, and Cuvier have trodden." Like them he appears hostile to the idea of a vital principle—resolves to confine his observations to vital properties—and feels no wish to draw aside the veil from Nature, and penetrate first causes. He disapproves highly of those physiologists who "suppose the structure of the body to contain an invisible matter or principle by which it is put in motion"—such as the *impetum faciens* of Hippocrates, the *archeus* of Van Helmont, and the *vital principle* of moderns. "Most of these terms, Mr. Lawrence observes, have long lain in cold obstruction amongst the rubbish of past ages; and the modern ones are hastening after their predecessors to the vault of all the capulets."

"By this language, his determined hostility to the vital principle is sufficiently evinced. In writing the last words, he seems to have actually embodied it in his fancy, to have thought of its life, its death, and of its grave, though not, we should hope, of the dark night, of the poison, and the dagger, that are said to have been present at the melancholy scene which once took place in the vault of all the Capulets.* In speaking of this principle, when his mind appears in a calmer mood, he says, 'It is compared to magnetism, to electricity, and to galvanism; or it is roundly stated to be oxygen. 'Tis like a camel, or like a whale, or like what you please.'† As in every shape, however, it appears to annoy him, it is no wonder, that, like Lucretius, when in horror at religion, he should feel an inclination to trample it under foot, and that to prevent his imagination in future from being haunted by so troublesome a spectre, he should labour to prove that it did not exist. In proceeding to his proofs, he says, 'We do not profess to explain *how* the living forces in one case, or attraction in the other, exert their agency.'‡ This is said in imitation of Blumenbach, and, he fondly fancies, in imitation of Newton also, who, in showing that the motions of the heavenly bodies follow the same law as the descent of a heavy substance to the earth does, contented himself with explaining the fact.§ Taking these two authorities for his example, he proposes to trace the phenomena of life and organization no farther than to certain laws, powers, properties, forces, and impulses, which, in imitation of his principal guide Cuvier, he chooses to call vital. 'Foiled (he says,) in our attempts to ascend to the origin of organized beings, we seek to inform ourselves concerning the real nature of the powers which animate them, by examining their composition, by investigating their texture, and the union of their elements. In them only can the vital impulse have its source and foundation.'‖

"* See Shakspeare's Romeo and Juliet, Act V. Scene iii."

"† P. 169." "‡ P. 165."

"§. 'In shewing that the motions of the heavenly bodies follow the same law as the descent of a heavy substance to the earth does, Newton explained the fact.'" P. 167.

"‖ Ibid. p. 142."

Had he added, like Cuvier, from whom this passage is literally translated,* though without any reference, that the texture and union in some measure owe their existence and continuance to the vital forces, or the vital impulse, he might then have inferred, that as they were mutually the source and foundation of one another, there could be no rational motive for tracing them to the origin of organized beings. But Cuvier thinking that also necessary, so does Mr. Lawrence, who, modestly declining either to think or to speak for himself upon the occasion, has no alternative but to follow and obey; and therefore, though his language may appear to proceed directly from himself, it is no more than a literal translation of that passage in which Cuvier† talks of the vital germ containing all the vital phenomena to be afterwards developed; of life proceeding only from life, and where he informs us, that the vital impulse said to have its source and foundation in the texture, as the texture had its source and foundation in the impulse, so this impulse is also to be considered as having at times a different origin, and to be an impulse not originating in the texture, but an impulse transmitted from the first parents of every species in an uninterrupted succession. On this new hypothesis, bodies are said to grow on bodies similar to themselves, from which they do not separate until they are sufficiently developed to act by their own powers, when, according to Mr. Lawrence, 'they grow by an internal power, and finally perish by that internal principle, or by the effect of life itself, exhibiting in their natural destruction or death, a phenomenon as constant as that of their first production.'‡

"This admission of an internal principle must strike the reader as somewhat remarkable, when that admission is by Mr. Lawrence, who scruples not to ridicule all those physiologists who suppose that any animal structure contains within itself an internal principle that puts it in motion." 336.

---

"* See the original, p. 329 of this Inquiry.

"† See Cuvier, vol. i. p. 6 and 7, and Mr. Lawrence's Lectures, p. 140, 141, and 142. See also Mr. Lawrence's advertisement, where the want of time, and a desire to print his Lectures as they were, are made the apology for omitting references."

"‡ P. 147. See also p. 145, 146."

"The idea that death is one of the necessary consequences of life, is, without acknowledgment, also borrowed from Cuvier. Upon the supposition that life is motion, Cuvier infers that it must cease like every other motion which is not in vacuo: "Il paroit même que la vie s'arrete par des causes semblables à celles qui interrompent tous les autres mouvemens connus, et que le durcissement des fibres et l'obstruction des vaisseaux rendroient *la mort une suite nécessaire de la vie*, comme le repos est celle de toute mouvement qui ne se fait pas dans le vide, quand même l'instant n'en seroit pas prévenu par une multitude de causes étrangères au corps vivant.'—Vol. i. p. 5.

"The opposite opinion that life is one of the consequences of death,

What then, Dr. Barclay asks, is the meaning which Mr. Lawrence attaches to *his* internal principle? He has told us, on the authority of Cuvier, that the vital impulse has its source in the texture and union of the elements which compose the structure—that life proceeds *only* from life—and that both life and vital impulse have been transmitted from our first parents by an uninterrupted succession. On the same authority, he has also told us that death is the necessary consequence of life itself—and lastly, brings forward Cuvier to tell us that "the idea of life is one of those general and obscure notions produced in us by observing a certain series of phenomena possessing mutual relations, and succeeding each other in a certain order:—that the vulgar, regarding these phenomena as the sign of a particular principle, have given to that principle a name, though in fact that name can only indicate the assemblage of the phenomena which occasioned its formation." "As to the question what assembled the phenomena?—Mr. Lawrence, before he presumes to reply, has to consult Cuvier, who is unable, however, to solve the difficulty." He only knows that they are assembled, and that they are held together by a link; but he knows as little of the link as of the cause of their assemblage. You may call this cause what you please, with this proviso, that you do not look on it with the vulgar, as a particular or vital principle—though both Cuvier and Lawrence have, oftener than once, in speaking of the cause of vital phenomena, used language that leads irresistibly to the vulgar idea of a separate principle contained within the structure, and putting it in motion. If the assemblage of vital phenomena, as digestion, circulation, nutrition, &c. forms this principle, as Cuvier states, how come these phenomena to be called *vital?*

"He seems not to reflect, any more than Cuvier, that the epithet vital in these cases can only imply, that they are the forces, properties, or powers, of a living being; and that, therefore, in annexing the idea of vitality either to a force, a property, or a power, we must first, though absurdly, suppose it personified, and then view it either as a distinct body or spirit. Of this absurdity, Mr. Lawrence himself, on certain occasions, seems to be aware; and on these occasions ascribes his living powers and his living properties to living bodies. So far, certainly, he is quite intelligible: but this question follows, How does it happen that his living bodies come to be

---

has obtained the preference with Buffon and Needham, whom we have seen maintaining with the ancients, that the corruption of one body is the generation of another. *Corruptio unius generatio alterius.*"

formed by their own properties? Here he has no answer to give: *he even acknowledges, that he has no hesitation in affirming that no connexion has been established in any one case between the organic texture and its vital power;* a most singular confession, at least from him. But this leads to another question: If no connexion has been established, how comes he to employ the expression organic texture and *its* vital power? Had he not as good reason for saying the vital power and *its* organic texture? This last expression might however seem to imply that the vital power was the cause of the texture; a supposition which is admitted only when he is accounting for the origin of the texture. In general we are given to understand that the functions are the offspring of the structure, or that the life is the result of the organization; and that the two are consequently connected as cause and effect, between which, it would seem, his experience does not warrant any idea of necessary connexion." 340.

We do not deem it necessary to follow our author any farther in his criticisms on Mr. Lawrence's observations on organization, cause or effect, or the mode of cultivating physiological science. Our readers have now pretty well seen that this section has not brought us *much* nearer to a knowledge of life than any of the preceding sections. We are, in fact, just where we started—unless it be that the farther we go in the inquiry, the darker is our path, and the more we become convinced of our utter ignorance!

Dr. Barclay concludes his critique on Mr. Lawrence with a lamentation and a eulogy conjoined.

"Had Mr. Lawrence only caught a portion of his (Mr. Hunter's) spirit, or a portion of the spirit of Harvey or Haller, and, in prosecuting physiology, dared to think for himself, regardless of Cuvier's or any other's creed; there are few, perhaps, and there have been few, whose ardour, industry, abilities, learning, and opportunities, would have entitled them to entertain such rational hopes of similar success." 348.

Cabanis.—This gentleman, who has written a large work on the relations between the moral and physical part of man's nature, has taken great pains to prove that the moral part has its origin in the physical—that all our ideas may be traced to sensation or to sensibility—and that these last can be traced no farther than organic structure and its functions. According to Cabanis, it was Democritus who first dared to conceive a system of the world founded on the properties of matter and the laws of motion. He represents this ancient Materialist as being found by Hippocrates very busy in dissecting the brains of animals, in order to unveil the mysteries of physical sensibility, and to discover the organs and causes which produce thought.

"· Hippocrate, appelé par les Abderitains, pour guerir Democrite de sa prétendue folie, le trouva disséquant *des cerveaux* d'animaux, dans lesquels il s'efforçoit de démêler les mystères de la sensibilité physique, et de reconnoître les organes et les causes qui produisent la pensée."

On turning, however, to the letter of Hippocrates to his friend Damagetus, we find that Cabanis has, with the greatest effrontery, *falsified the text.* In the letter there is no allusion whatever to this unveiling of mysteries in the search after the organs of thought. Democritus, poor man, was merely dissecting animals for the purpose of finding out the nature and seat of *bile*, which he conceived to be the cause of madness! " Nam animalia hæc, quæ vides, inquit, hujus gratia reseco, *non quod odio habeam opera Dei*, sed *bilis*, naturam ac sedem quærens. Nosti enim quod hæc furoris hominum causa est, ubi nimium redundarit."* This is a pretty specimen of Cabanis's fidelity! We shall, however, exhibit another. According to this gentleman, it was Locke who first gave development to this idea of Democritus, by establishing on clear and direct proofs, *that all our ideas are derived from the senses!* Now let us see what Locke says on this point. That philosopher, it is true, asserts that all our ideas are derived *either* from sensation or *reflection;* but sensation and reflection, in his language, are words of different import—denoting distinct sources of ideas.

" ' Our *senses*," he observes, " conversant about particular sensible objects, do *convey into the mind* several distinct *perceptions* of things, according to those various ways, wherein those objects do affect them: and thus we come by those ideas we have of *yellow*, *white*, *heat*, *cold*, *soft*, *hard*, *bitter*, *sweet*, and all those which we call sensible qualities; which, when I say the senses convey into the mind, I mean, they from external objects convey into the mind what produces there those *perceptions*. This great source of most of the *ideas* we have, depending wholly upon our senses, and derived by them to the understanding, I call Sensation.

" ' The other fountain, from which experience furnisheth the understanding with *ideas*, is the *perception of the operations of our own minds* within us, as it is employed about the *ideas* it has got; which operations, when the soul comes to reflect on, and consider, do furnish the understanding with another set of *ideas*, which could not be had from things without; and such are *perception*, *thinking*, *doubting*, *believing*, *reasoning*, *knowing*, *willing*, and all the different actings of our own minds, which we, being conscious of, and observing in ourselves, do from these receive into our understandings as distinct *ideas*, as we do from bodies affecting our senses. This

* Vol. II. p. 917. Vander Linden's Edition, 8vo.

source of *ideas*, every man has wholly in himself: and though it be not sense, as having nothing to do with external objects, yet it is very like it, and might, properly enough, be called internal sense. *But, as I call the other Sensation, so I call this Reflection; the ideas it affords being such only as the mind gets by reflecting on its own operations within itself.*' "* 358.

With what confidence, Dr. B. asks, are we to rely either on the accuracy or veracity of Cabanis, who thus imputes opinions to Locke which he never held, "and who, falsifying the letter of Hippocrates to Damagetus, imputes to Democritus a course of studies of which that philosopher seems never to have dreamed." In short, had Cabanis comprehended the meaning of Locke, and possessed the candour to represent him fairly and honestly, he would rather have said that Locke was the first, or among the first, who fully exposed the *falsehood* of that old scholastic maxim—" *Nihil est in intellectu quod non fuit prius in sensu.*"

Cabanis having made some use of *physiognomy*, in his hypothesis, Dr. Barclay takes occasion, in this place, to offer some keen, but not illnatured, strictures on it, as well as phrenology or craniology. In respect to the first, or physiognomy, Dr. B. justly observes that the superstitious credulity of mankind is always ready to welcome and embrace such a science, (if it deserve that name,) not only in opposition to reason, but in opposition to the numerous facts which belie its predictions. Our author means not to deny indeed that there are many external appearances in the form, proportions, and attitudes of the body, by which we are led, partly by instinct, and partly by previous associations founded on experience, at once to form some idea of the health, temper, and dispositions of the individual. But he properly objects to the pretension of forming any correct idea of a man's private or peculiar character from these alone, without knowing something of his actions, previous education, opinions and prejudices, or of those arts which he may have acquired in counterfeiting various outward expressions that bespeak neither the feelings of his heart, nor the thoughts of his head. In short, the absurd attempt to judge of the whole of a man's character by merely examining a part of his body, his hands, or his head, has been so invariably the practice of physiognomists, that physiognomy now denotes little more than a species of fortune-telling.

Cabanis alludes to the old opinions respecting the four humours, to show that the ancients (as well as the moderns) had frequently observed how much the nature of the several

---

* Essay on Understanding, Vol. I. p. 68. Lond. 1784. 2 vols. 8vo.

passions, affections, and thoughts was dependent on the state of the body. Now there can be no rational objection to this general conclusion, that the body modifies the vital phenomena. It is only when Cabanis presumes to assert that the body not only modifies, but is really the sole *cause* of these phenomena, that we should be disposed, with Dr. B. to enter our dissent, and to deny that such a conclusion is warranted by his premises, or by any observations which he has made. We shall make no apology for here inserting a rather long extract from Dr. Barclay, since we conceive that it exhibits enlightened views, and at least rational conjectures.

" Suppose that, agreeably to a hypothesis not only of Robinet, but of other philosophers, there are two worlds, a visible and an invisible, and that there are many species of beings belonging to the latter, which, endowed with the power of organizing matter, form to themselves specific structures adapted to their several capacities and energies; would it not follow, on this supposition, that these beings, in constructing their systems of material organs, or in employing these organs afterwards, would at least be regulated by some laws peculiarly their own, and exhibit phenomena, through the medium of such organs, which could not with any propriety be ascribed to any of the substances composing the material or visible world? Considering, besides, that those systems of material organs would still be subjected to the general laws of the visible world, would there not necessarily be a reaction between those systems and the invisible beings within them? and until the relation between them should be dissolved, would not the systems continue subjected to two distinct species of laws at the same time; one species peculiar to the invisible beings acting from within; and another species peculiar to the visible or material world acting from without? Now, that there is some invisible agent in every living organized system, seems to be an inference to which we are led almost irresistibly. When we see an animal starting from its sleep, contrary to the known laws of gravitation, without an external or elastic impulse, without the appearance of electricity, galvanism, magnetism, or chemical attraction: when we see it afterwards moving its limbs in various directions, with different degrees of force and velocity, sometimes suspending and sometimes renewing the same motions, at the sound of a word or the sight of a shadow, can we refrain a moment from thinking that the cause of these phenomena is internal, that it is something different from the body, and that the several bodily organs are nothing more than the mere instruments which it employs in its operations? not instruments indeed that can be manufactured, purchased, or exchanged, or that can at pleasure be varied in form, position, number, proportion, or magnitude; not instruments whose motions are dependent upon an external impulse, on gravity, elasticity, magnetism, galvanism, on electricity or chemical attraction; but instruments of a peculiar nature, instruments that grow, that are moved by the will, and which can be regulated and kept in repair by no agent but the one for which they were primarily destined; instruments so closely related to that agent, that they cannot be injured, handled, or breathed upon, approached by cold, by wind, or by rain, without exciting in it certain sensations of pleasure or of pain; sensations which, if either unusual or excessive, are generally accompanied with joy or grief, hopes or alarms: instruments, in short, that exert so constant and powerful a reaction on the agent that employs them, that they modifiy almost every phenomenon which it exhibits, and to such an extent, that no person can confidently say what would be the effect of its energies if deprived of instruments; or what would be the effect of its energies, if furnished with instruments of a different species, or if furnished with instruments of different materials, less dependent on external circumstances, and less subjected to the laws of gross and inert matter. That logician therefore, that moralist, that divine, or that metaphysician, who overlooks the reaction of the organs, and proceeds to treat of the animating principle, as a thing unbodied, or at least not encumbered with material organs, is guilty of as unwarrantable an assumption, as that anatomist, surgeon or physician, who, considering only the reaction of the organs, most illogically concludes that they not only modify the energies of the animating principle, but are even the causes of its existence." 372.

Dr. Barclay observes that the voluntary organs are not restricted to any specific modes of operation—the human hand is not limited to acts of beneficence or cruelty. It is equally subservient to all instincts, appetites, and passions. An organ thus employed in such a variety of different offices, and executing each with promptness and precision, might lead the unwary to suppose it composed of a great variety of subordinate organs, corresponding with its different duties. Such conclusion would not follow, however, in reasoning analogically from the works of *Nature;* for although the hand is constructed of many dissimilar parts, it is *not* on the principles of a time-piece, whereof each index requires a distinct apparatus. In the human hand all the parts are observed to combine in each operation—and the varieties of these operations are not so much owing to the number of parts, as to the varieties of their combinations—these last being almost incalculable.

" Taking the hand then as a specimen of the works of nature and of animal structure, and thence reasoning on the principles of analogy, with respect to the brain, ought we not to infer, that all the parts of which it is composed may also combine in a similar manner, and be concerned in every phenomenon which has been ascribed to it?" 374.

The phrenologist, it is true, cannot consistently draw this conclusion, imagining, as he does, that each specific phenomenon, or series of phenomena, is the effect of a specific faculty, and that each faculty has a specific system of organs by which it perceives, conceives, imagines, and remembers, in a manner peculiar to itself.*

If the metaphysicians, as Dr. Barclay wisely remarks, have long been censurable for personifying, as it were, what they call powers and faculties, and viewing them as connected loosely, like a bunch of keys hung upon a ring, or engrafted branches deriving their nourishment from the same stock, the phrenologists seem inclined to push this hypothesis still farther, not only increasing the number of faculties but the number of organs. Besides the organs of sense long known to anatomists, they have found or fancied no fewer than thirty-three species of organs or systems in the human brain—and these all in pairs: a pair for each species of propensity, a pair for each species of sentiment, a pair for each species of knowledge, and a pair for each species of reflection. If you ask for ocular demonstration, you are told these organs are indicated by thirty-three modifications observed in the form of the skull, occasioned, of course, by a corresponding number of modifications in the brain. Yet on opening the skull, and examining the surface of the brain, where these organs are said to be situated, it requires no small share of creative fancy to see any thing more than a number of almost similar convolutions, *all* composed of cineritious and medullary substance nearly in the same proportions, and *all* exhibiting as little difference in their form and structure as the convolutions of the intestines—" nay, all, when unfolded, according to Spurzheim, in cases of *hydrocephalus internus*, presenting but one uniform web of cineritious and medullary matter." No phrenologist has ever yet attempted to draw the line of demarcation between these organs; but supposing they were divided, and presented promiscuously and detached, to a craniologist, would he venture to distinguish merely by their form and structure, an organ of propensity from an organ of sentiment—an organ

" * 'The faculty of tune, for example, perceives, conceives, imagines, and remembers, melody alone; the faculty of causality, perceives, conceives, imagines, and remembers, ideas of necessary consequence and nothing else. One faculty, like one branch, comes to maturity sooner than another; one may be strong, and another weak, in the same or in different individuals; one may decay or become diseased, and the others remain vigorous; and all this in consequence of each faculty having a distinct and specific organ."—*Illustrations of Phrenology*, p. 51.

of sentiment from an organ of knowledge—or an organ of knowledge from an organ of reflection? He would be a hardy craniologist if he did! But although it is so difficult to recognize these organs when the skull is removed, yet it appears that they are so very prone to affect conspicuous situations, and obtrude themselves on the notice of the senses, that there is not any visible part on the crown of the head, on the frontal bone, on the occiput or temples, where, according to the craniologist, they do not exhibit, even through the hardest and the thickest skulls, undeniable proofs of their actual presence. Dr. Barclay shrewdly and somewhat jocosely asks, "is it then, in order to be always within the sphere of physiognomic and phrenological investigation, that they equally avoid the central parts of the cerebral substance?" We fully agree with our judicious author in the following passage?—

"To the observations made by phrenologists on the forms of the head, as indicative of the several powers and capacities of the animating principle, if made with sufficient caution and accuracy, and if the relations which they wish to demonstrate can be fairly established upon the broad principles of induction, there can be no rational objection. Their supposed organs rest upon a quite different foundation: not being demonstrable in form or in structure, they must ever remain the mere offspring of a hypothesis; and of a hypothesis that may be disproved by what is termed a *reductio ad absurdum*.." 379.

Dr. Barclay pays a just compliment to the learning, candour, and liberal sentiments of Dr. Spurzheim, Sir George Mackenzie, and Mr. Coombe, with whom he is personally acquainted, and who, he feels conscious will not be offended at these remarks.

"Although," says Dr. Barclay, "I think that in many instances their premises are far from supporting their conclusions, and that the latter too frequently rest on the former, like a pyramid on its apex, or a broad superstructure upon a narrow and tottering basis; yet, to their writings I acknowledge myself to be much indebted for several new and important views which they have suggested, and therefore will never subscribe to the sarcasm, that what is true in their doctrines is not new, and that what is new in them is not true. Though evidently inclined to favour physiognomy, yet they seldom fail to caution their readers against its extravagancies: a warning, I suppose, which they have found to be highly requisite: but although, like physiognomists, they be apt to ascribe more influence to the organs than is strictly warrantable, in many cases imagining them to command where they only obey, they never once question the existence of an internal animating principle, nor, like Cabanis, endeavour to maintain that whatever is moral and intellectual in man is merely the consequence of organic structure." 381.

Dr. Barclay's short section on the share which *chemical affinities* have in the organization of animals, need not occupy any space here. No man of common sense, or common observation can, for a moment, confound together vital and chemical phenomena. It is sufficient to say that, Chaptal and Thomson deny the connexion, affinity, or identity.

This brings us to the third and last chapter of the work, exhibiting a sketch of the opinions of some eminent characters, ancient and modern, who have supposed a living internal principle distinct from the body, and likewise the cause of its organization. The writers noticed on this side of the question are, Aristotle, Harvey, Willis, Hunter, Abernethy, Deleuze, Grew. Over these we must make rapid strides, as our limits are drawing towards a close.

Aristotle. This powerful genius, after enumerating the various opinions entertained by others concerning the soul, gives us his own opinion. He pronounces it to be a substance belonging to the order of real entities—the first *entelecheia*, or primary principle of action in a natural organized body, susceptible of life, totally different from that which is known to move an automaton or artificial organized body. He then goes on to explain this definition more clearly. Thus, in considering what holds the fabric of the universe together, and forms out of the discordant elements a harmonious whole, he infers, from analogy, that it must be something similar in kind, to that which holds together an organized body—namely, a principle of life; and that this principle, from the appearance of order and design displayed in the universe, must also have intelligence, and have existed before the elements or matter. This supreme animating principle, which he denominates Theos, or God, is invisible to mortal eyes, and can only be known through his works—who is not incorporated with any of the elements, but who resides apart and alone extending his influence to all things on earth and elsewhere.

"Besides this supreme animating principle, the author and preserver of all, there are many others, according to Aristotle, of an inferior and subordinate nature, which, by delegated powers, organize the bodies of animals and plants, so that all organized bodies whatever are to be considered as constructed by, and constructed for their animating principles; which, like the great animating principle, from being invisible to mortal eyes, indicate their existence, their energies, and their species, only through the medium of the structures which they form. Now, of these structures they are not only the efficient causes, but, in his opinion, the formal and the final; the causes of their motions, growth, and nutrition; the causes which give them a character and form; the causes on whose account they exist; and even the causes of their being afterwards liable to corruption, as nothing is corrupted but what has been nourished, and has some time or other partaken of life." 432.

It is not a little singular that Aristotle, after admitting and endeavouring to prove, that every soul organizes the body, and constructs for itself peculiar organs suited to its faculties, should yet be inclined to believe, that souls are indebted for their existence to the bodies which they have formed, and must consequently perish with them. This opinion is still more singular, when we find him maintaining that the soul is not affected by age—that the dimness of sight, and the effects of sickness and ebriety, are not the consequences of any change in the state of the faculties, but only of a change in the state of the organs—in short, that where the intellectual and contemplative faculties seem to be impaired, we are only to infer that, some organ of the body is diseased, the faculties themselves being still entire, from their participation in the nature of the Deity. He thinks, however, that the Nous, or intellective and contemplative faculties are separable from the other faculties and from the body, and, in conjunction, are capable of maintaining an independent existence.

Harvey. After an active life spent in study, observation, and experiments, Harvey came to the same conclusion with Aristotle, that all animals and plants owe their form, structure, growth, and power of resisting putrefaction, to an animating principle which exists and operates before any organ of the structure can be formed. As the plan on which this principle appears to act unconsciously, implies much power, intelligence, and foresight, Harvey ascribes it exclusively to him who formed the universe itself, and who appears to be every where present as the superintendent and director. But Harvey, like Aristotle, is not free from inconsistencies. Warped by his favourite hypothesis respecting the blood, he imagined that this fluid is formed and moved before any vessel or any organ of motion exists—that in it, and from it, not only do motion and pulsation originate, but animal temperature, the vital spirit, and even the principle of life itself—that it is the first thing that lives, and the last that dies. With all the inconsistency of the philosopher, he asserts that, the blood appears to differ in nothing from the anima, and that what he had already conceived to be a principle distinct from the blood, appears now to be no more than an act of that fluid! To support this fanciful hypothesis, he appeals to the sacred writings, where the blood is symbolically denominated the life of the flesh. With equal propriety might he have appealed to the figurative language of Virgil, where a purple life and a purple death are synonymous expressions. "Purpuream vomit ille animam."

We shall pass over the lucubrations of Willis, "who indiscriminately rests his opinions on hypotheses and facts," in the most cursory manner; since, we fear, we have already made considerable demands on the patience of our readers. He believes, with Aristotle, that each individual animating principle organizes the body in which it resides, and always constructs it to suit the specific and peculiar faculties with which it is endowed, and which it is afterwards to exercise. He agrees, also, with Harvey, in supposing that this principle resides in the blood or circulating fluids, denominating it the corporeal soul, and describing it as of an igneous nature, and formed of such particles as the body itself, but of a subtle and refined kind.

Hunter. The mind of this gentlemen was not stored with much of the riches of literature. Most of his opinions, though thought by himself and perhaps his friends, as original, are neither new nor uncommon. Thus, having inferred from experiment, not only that a prolific egg has the power of preservation, or a principle of life, but, that organization and life do not depend, in the least, on each other—that organization may arise out of living parts and produce action, but that life never can arise out of, or depend on organization, he seems anxious to secure to himself the honour of these discoveries, though Harvey performed the same experiments a hundred years before, and drew all his conclusions, excepting that one, in which organization and life are made not to depend in the least on each other—a conclusion which, unfortunately, is contradicted rather than warranted, by the premises on which it is made to rest. He appears, also, to have laboured under the singular delusion, that he himself was the first person who had ever dreamed of the vitality of the blood; and yet, the observations and experiments which he made with that view, are neither so numerous nor so forcibly stated, as those which had previously been made by Harvey. After all, he seems never to have entirely divested his mind of the idea of a living power in the solids. What he chiefly contends for is, that there is a principle of life in the blood as well as in the solids—a principle which he calls the *materia vitæ*, and which, principally, he says, composes the brain. On this hypothesis, the brain is the *materia vitæ coacervata*, the nerves chordæ internunciæ, and the like matter diffused through the blood and body in general, the *materia vitæ diffusa*. But he abides not long by this opinion; for he soon afterwards thinks, that this cerebral matter is not so essential to life as the blood—that life commences even in the chyle—

while these fluids, on another new hypothesis, are supposed to acquire their activity in the lungs! It is not surprizing that, from such a chaos of inconsistent and contradictory ideas, he was unable to draw any rational conclusion. In searching for the principle of life, on the supposition that it was the property of something visible, he has, fruitl ssly enough, looked for it in the blood, the chyle, the brain, the lungs, and other parts of the body—but not finding it in any of them exclusively, he then concluded, that it must be a consequence of the union of the whole, and depend upon organism—the precise conclusion which has still more recently been brought up again by Mr. Lawrence and others. But, to this conclusion, Mr. Hunter could not long adhere, after observing, that the composition of matter does not give life, and that a dead body may have all the composition it ever had. Last of all, he draws the true, or at least the candid conclusion—*that he knows nothing at all about the matter.**

Mr. ABERNETHY. We have always looked upon the theory of this gentleman as not a less decided doctrine of materialism than that of Mr. Lawrence; and, to us it appears perfectly evident that, had Mr. Lawrence delivered his opinions without dogmatism, without irony, and without sneers at religion,—had he delivered them as philosophical *conjectures*, (and he ought to have known that they were but conjectures a hundred times conjectured,) they would never have involved him in the sea of troubles which now surround him. It is, we repeat it, the *manner* and not the *matter*, which is more objectionable in Mr. Lawrence's than in Mr. Abernethy's writings.

Mr. A. has adopted *that* opinion of Mr. Hunter, which makes the principle of life independent of organization—a something *superadded* to the organized structure. "In defending this opinion, however, he supports it with much modesty and candour, and no where attempts to enforce it by sophisms or dogmatical assertions."† We are now to inquire on what evidence is founded the supposition, that life is *superadded* to organized structure.

"If the organized structures of the first parents of every species were formed in the first place, and then their vital principles superadded, can it be supposed that this is the manner in which the structures of any of their progeny are now formed? Has any physiolo-

---

* "But mere composition of matter does not give life; for the dead body has all the composition it ever had: *life is a property we do not understand.*"—*Hunter on the Blood, p.* 90.

† *Barclay, p.* 485.

gist ever observed an animal or a plant whose visible structure had not been formed by successive processes of organization? or ever seen a process of organization which did not indicate the previous existence of a vital principle? Nay, were we to trust either to the evidence of our sense or our reason, should we not say that, in our days at least, the structure appears to be rather superadded to the vital principle, than the vital principle superadded to the structure? To repeat an observation formerly made, it is upon this hypothesis alone that Cuvier and other naturalists have concluded, that plants and animals which happen to be found in a fossile state had formerly been alive: but the question occurs, what gave them life? or what preceded their organization? Mr. Abernethy supposes a subtile substance of a quickly and powerfully mobile nature, which pervades every thing, and appears to be the life of the world; a substance which he thinks may be considered as a distinct and active principle, not confounded with intelligence of any kind," 488.

This notion he ascribes to Mr. Hunter; but every body knows that, there is scarcely a notion more ancient than this *anima mundi*, diffusing life and motion through the universe, though devoid of intelligence itself. This vital principle, Mr. A. seems to attribute to electricity. "Thus," says he, "if the vital principle of Mr. Hunter be not electricity, at least we have reason to believe it is of a similar nature, and has the power of regulating electrical operations." Yet, it is singular, as Dr. Barclay shrewdly observes, that it did not occur to Mr. Abernethy, that the power of regulating electrical operations belongs more properly to an electrician than to electricity—to the agent directing, rather than to the material directed. But how can it be conceived that, this unintelligent and unconscious material, (whatever it may be,) should so vary its modes of operation, as to construct the almost innumerable species of organized forms to be found in the animal and vegetable kingdoms—each constructed on a plan, and each plan different from another? Is it not far more natural to suppose, that every animal has a specific organizing principle, with specific powers to organize, to feel, and to be guided by specific instincts? and, that the individuals of the human species have, in like manner, a specific principle, not only to organize and to feel, and to be guided by specific instincts, but even to reason?—Every thing, indeed, seems to support the probability of this opinion, though some speculative physiologists would have us believe that, one subtle unconscious substance is capable of beginning, or if not of beginning, of carrying to perfection, when once begun, all the various species of organisms; and, that a common sensitive principle, in the same way, is sufficient to account for all kinds of sensations.

"But must nature be compelled to accommodate herself to their conceptions and to their inclinations? Is not her right to dictate to them better than their right to dictate to her? When God said, Let there be light, there was light; but can they pretend to any such power? Certainly not: they can indeed, as often as they choose, call spirits from the vasty deep, and so can we, or so can any man; but will they come when either they or we do call for them?*" 494.

The plain truth is, that the way in which the supreme Being has bestowed upon us, and upon every species of animal, that singular power by which we can move, by an act of the will, those masses of matter, which constitute our bodies, must to us remain for ever inexplicable. But the fact may enable us to form some idea how the Creator, by a mere act of *His* will, could call the universe into existence, and still uphold it without either labour or fatigue.

"Might we here be permitted to form a conjecture on a subject which so far exceeds our comprehension, to us it might seem, when he called into existence those spirits which inhabit organisms, as if he had said, You inferior spirits require organisms of gross materials to hold communication with the visible and tangible objects around you; and as my will is every where obeyed throughout the extensive regions of space, I have willed that all of you construct organisms suited to the faculties with which I have endowed you; but as I have not bestowed upon you faculties to comprehend their mode of formation, or their mode of acting, I have ordained, that when you shall will, your organisms shall obey, though how they obey you shall never know, being creatures of too limited intelligence to comprehend the wisdom, the power, the ways, and the doings of the Most High." 499.

Our author's section on animal magnetism, and particularly the system of *Deleuze*, (or, without meaning a pun, the system of *delusion,)* we shall pass over in a very cursory manner. We are surprized, indeed, that Dr. Barclay should have taken the pains to wade through such a sea of absurdities, with the expectation of finding any thing else than the dreams of credulous, crack-brained, or designing men. He has waded through it, however, and he asks, whether the several phenomena of animal magnetism, *allowing that they are stated correctly*, establish any general fact with which we were not previously acquainted? Have not most of us, he observes, felt, when there was nothing to arrest our attention but insignificant nods, shrugs, waving of hands, *or tiresome drawling, monotonous tones of an uninteresting, long-winded*

---

* See Shakspeare's King Henry IV. part i. act iii. sc. 1.

*orator, that we have irresistibly been compelled to sleep?** Might we not have learnt from daily experience, that the voluntary motions of our bodies are under the direction of some vital principle within us? "and that this principle and our voluntary motions may also be brought under subjection to another principle of the same species?" Do we not see the voluntary motions not only of one or two, but even of thousands in fleets and armies, in a great measure regulated by the will of one individual? Have we not observed that will communicated to thousands almost instantaneously by a word, by a sound, or a visible sign, and afterwards operating through their hopes or their fears, their astonishment, or their despair, so as to affect their thoughts while awake, and their dreams while asleep?† These facts, by their familiarity, are passed unnoticed, while we wonder at the phenomena of animal magnetism that are nothing more than those which we every day see.

After giving some short account of the opinions of Grew, as contained in his Cosmologia Sacra, but which need not detain us here, Dr. Barclay offers a brief "summary view," of what our readers have seen more in detail in the course of this article. We do not deem it necessary therefore to make any recapitulation of those details here, but shall notice the concluding portion merely of Dr. Barclay's summary.

After remarking on the inadequacy of all those explanations which have been offered by philosophers, and which we have amply delineated, Dr. Barclay adverts particularly to the insurmountable difficulty which the Sceptics have found in explaining how the *first parents* of the different species of animals might possibly have been formed. In fact, the Materialists studiously shun this question, and that under the pretence that such inquiries might lead them into the *regions of fancy*—regions, forsooth, in which they are not at all disinclined to roam upon all other occasions! They saw distinctly that first parents could not be formed, either in the womb or in any other organ of a parent, at a period when no parent existed—in short, they saw the in-

---

* We suspect Dr. Barclay has a sly hit at some other than magnetic orators in the latter part of this passage.

† Every body knows the mental contagion, for instance, which spread so rapidly through the hospital at Haerlem, and which, from the sight of a single individual falling into fits, diffused itself in the form of epilepsy through most of the young persons in the house, and was afterwards as suddenly checked by Boerhaave without employing other means than that of inspiring the patients with the dread of much greater horrors, if they ever again submitted to such fits.

superable difficulties which they had to encounter, if they allowed their fancies to ascend to the origins of things—and to avoid this embarrassment they chose to assert that all events move in a circular, and that all species of plants and animals may have been eternal!—imagining that by this contemptible subterfuge they would evade the obvious question—"how came the first parents to be formed in a manner so different from that by which their progeny is formed now?" We believe, with Dr. Barclay, that the account of our first formation, as given by Moses, is the best that ever was promulgated, whether that writer was inspired or not. The cause which he assigns is an omnipotent, omniscient, omnipresent Being, invisible, self-existent, and eternal, to whose will the whole universe is subjected more thoroughly and completely, though not more inconceivably, than our bodily organisms are subjected to our wills. Moses says, and so think we, that this supreme Being did create man, as well as the earth he treads on, by the fiat of his will, giving him the dominion over other animals, and endowing him with the power of judging between what is morally right and wrong—with the faculty of reason—with the capacity of tracing phenomena to invisible causes; and ultimately to the creator himself. The original mandate, "be fruitful and multiply, each after its kind," was obeyed at the beginning, and it is in force to this day. It was issued to animals and to man, by the same Deity.

"Nor let man envy the honour thus bestowed on his fellow creatures. He has obtained the dominion over them: so let those who are conscious of this marked distinction, who are capable of tracing the origin of their species to the first of causes, and who feel that they are under the protection of an omnipotent, omniscient omnipresent being, self-existent, benevolent, and just, be therewith content, and congratulate themselves that they are not reduced to that low and degraded state of some modern physiologists, who with all their efforts, have never been able to trace their origin beyond some gross collections of matter, some occult qualities, or some unknown chemical affinities of mud or atoms; and who, as to religion, have only to console themselves with the thought, that they are at least as far advanced as the Caffres, the Hottentots, and the untutored savages of Brazil." 531.

Thus concludes Dr. Barclay's work—a production equally creditable to his head and his heart. The motives which have led him through such toilsome researches are unquestionably of an honorable and commendable kind, for they unequivocally point to the welfare and happiness of mankind at large, by inculcating veneration for the Deity, respect for themselves, good-will towards their fellow creatures, and charity towards the whole of animated nature. But the peculiar or local tendency of the work, we conceive, is to shew the rising generation of the profession the folly of prying into first causes, by pourtraying the wild, contradictory, and ridiculous theories into which the most eminent philosophers of all ages have been led, when attempting to unravel those mysteries which Nature, or Nature's God has thought proper to veil from human eyes. It was with the view of furthering the objects of Dr. Barclay's work that we have entered so extensively into its analysis, and endeavoured to convey a pretty accurate idea of its prominent features to our readers. We have little to add to those remarks which we have occasionally made in the course of the article, now protracted beyond what we originally calculated on. The psychological turn which anatomical and physiological investigations have lately taken, will, we fear, be injurious in many ways. These investigations will lead us from the direct path of our own proper studies, and tend to unsettle not only the minds of professional men, but of the vulgar themselves. In the present intellectual state of society it is as utterly impossible to have one set of opinions for the high and another for the low, as to have two suns to see by, two atmospheres to breathe by, or two kinds of sensorium to feel by. "For opinions," as a modern author justly observes,* "like showers, are generated in high places, but invariably descend into low ones, ultimately flowing down to the people, as the rains unto the sea." It is quite evident that the *doubt* of a future state of existence must be quickly followed by a *denial* of that state; and it is difficult to conceive what would be the effects of a general revolution in the minds of men respecting this momentous question. We cannot but think that, in such a case, all that endears us to our fellow men, and all that exalts us above the beasts of the field, must be swallowed up in the paltriness of the present and nothingness of the now! If a few sceptical philosophers have exhibited examples of virtue, honour, charity, and other christian virtues, we must recollect that all men are not philosophers, and that when the belief of a future state of existence was thrown off by the multitude, in the French revolution, all the dark traits of human nature instantly became prominent, and man proved himself (without the curb of religion or morality) to be "more fierce than tygers on the Lybean plain." Gibbon, when quaffing noyeau, and ridiculing the doctrine of a future existence, was asked what would pro-

* Mr. Coulton bably be the effects of this scepticism on society in general. His reply was this:—"the doctrines we are now discussing are, like the liqueur we are now drinking;—safe and even pleasant to us, who know how to use, without abusing them;—but dangerous, deleterious, and intoxicating, if either the liqueur or doctrines were broached in the open streets, or exposed to the discretion of the mob." Experience has now taught us that though bolts and bars may keep noyeau from the multitude, they are quite inefficient in confining sceptical doctrines, which are too volatile for such imprisonment. It may be urged, (as it has often been urged,) that the promulgation of truth can never be ultimately injurious to society. But how are we to ascertain the *truth* of these doctrines to which we are objecting? They are but speculations, and whether true or false, their promulgation is attended with bad effects in the mean time. We have often wondered how medical men, who daily witness the benign influence of religion in mitigating the miseries of sickness, and disarming the terrors of death, can bring themselves to broach doctrines subversive of all those consolations which the afflicted derive from the belief of a superintending Deity, and another and better world! We cannot but think that this wanton trifling with the feelings, the hopes, and the fears, of so large a portion of mankind, is little indicative of philanthropy in the breast, or good sense in the head, of the writer. It has been well observed that—"in literature our taste will be discovered by that which we give, and our judgment by that which we withhold." It would be for the benefit of society, and the credit of the medical profession, if such of its members as held sceptical opinions were to keep them confined within their own bosoms till the multitude are as great philosophers as themselves, and as capable of discussing metaphysical subjects.

God forbid that we should be advocates for fettering the human intellect, and checking the liberty of discussion. But while indulging ourselves in the full freedom of *thought*, we have no right to issue *words or writings* that may be prejudicial to the community of which we are members, and to which we owe our safety. There are many and cogent reasons why the medical philosopher should avoid, as much as possible, those metaphysical discussions that involve religious or political tenets. By the profligate, licentious, and factious portion of society (no inconsiderable portion) his speculations will be adopted only to turn them to the worst of purposes. By the fanatical portion, and by the intolerant or crafty part of the priesthood, they will be converted into engines of persecution against himself—while by the truly philosophic portion of society (which is comparatively small) the *promulgation* of these doctrines will be condemned, even if the principles they contain should be embraced. If indeed we look back into the records of the past, we shall find that an absolute freedom in discussions involving religion, morals, and politics, never yet existed in any age or country. It is one of the dreams of modern philosophy. The superstition of the Lacedemonians prohibited all enquiry on the subject of religion—and in Athens, the ancient city of intellect, the lives of Æschylus, Socrates, Alcibiades, and many others, demonstrated that neither genius, learning, courage, nor the softer virtues, could screen their possessors from the persecutions of an implacable priesthood. In Rome it was *toleration*, not freedom. In Europe, wherever freedom has been allowed, licentiousness has followed. We would therefore recommend to our brethren a close adherence to the legitimate subjects of our own science, (where unhappily there is but too much to be yet accomplished) leaving all metaphysical speculations to those whose lives are spent more in the closet than in society—in contemplation than in action —in ascertaining the attributes of the soul, rather than in watching the diseases of the body.

